# Relationship between Sleep Disorders and Health Related Quality of Life—Results from the Georgia SOMNUS Study

**DOI:** 10.3390/ijerph15081588

**Published:** 2018-07-26

**Authors:** Nato Darchia, Nikoloz Oniani, Irine Sakhelashvili, Mariam Supatashvili, Tamar Basishvili, Marine Eliozishvili, Lia Maisuradze, Katerina Cervena

**Affiliations:** 1 Research Center—T.Oniani Laboratory of Sleep–wakefulness Study, Ilia State University, 0162 Tbilisi, Georgia; nikoloz.oniani@iliauni.edu.ge (N.O.); irine.sakhelashvili.1@iliauni.edu.ge (I.S.); s.mariam247@gmail.com (M.S.); tamari.basishvili@iliauni.edu.ge (T.B.); marine.eliozishvili@iliauni.edu.ge (M.E.); lia.maisuradze@iliauni.edu.ge (L.M.); 2 Sleep Medicine Center, Division of Pneumology, University Hospital of Geneva, Chêne-Bourg, 1225 Geneva, Switzerland; katerina_cervena@yahoo.com; 3 Sleep Medicine Center CENAS, Les Acacias, 1227 Geneva, Switzerland

**Keywords:** health-related quality of life, sleep quality, insomnia, sleep apnea, daytime sleepiness, Georgia

## Abstract

The extent to which sleep disorders are associated with impairment of health-related quality of life (HRQoL) is poorly described in the developing world. We investigated the prevalence and severity of various sleep disorders and their associations with HRQoL in an urban Georgian population. 395 volunteers (20–60 years) completed Pittsburgh Sleep Quality Index, Epworth Sleepiness Scale, STOP-Bang questionnaire, Insomnia Severity Index, Beck Depression Inventory-Short Form, and Short Form Health Survey (SF-12). Socio-demographic data and body mass index (BMI) were obtained. The prevalence of sleep disorders and their association with HRQoL was considerable. All SF-12 components and physical and mental component summaries (PCS, MCS) were significantly lower in poor sleepers, subjects with daytime sleepiness, apnea risk, or insomnia. Insomnia and apnea severity were also associated with lower scores on most SF-12 dimensions. The effect of insomnia severity was more pronounced on MCS, while apnea severity—on PCS. Hierarchical analyses showed that after controlling for potential confounding factors (demographics, depression, BMI), sleep quality significantly increased model’s predictive power with an R^2^ change (ΔR^2^) by 3.5% for PCS (adjusted R^2^ = 0.27) and by 2.9% for MCS (adjusted R^2^ = 0.48); for the other SF-12 components ΔR^2^ ranged between 1.4% and 4.6%. ESS, STOP-Bang, ISI scores, all exerted clear effects on PCS and MCS in an individual regression models. Our results confirm and extend the findings of studies from Western societies and strongly support the importance of sleep for HRQoL. Elaboration of intervention programs designed to strengthen sleep-related health care and thereof HRQoL is especially important in the developing world.

## 1. Introduction

Current evidence indicates that sleep disorders may contribute to the growing health burden in modern societies. Insufficient and poor-quality sleep represent high-risk factors for health outcomes such as cardiovascular disease, cognitive impairment, and metabolic dysfunction; poor sleep has also been associated with risk-taking behavior, accidents, increased mortality rates, and diminished quality of life [1,2,3]. The importance of sleep for promoting health, quality of life, and safety has been well recognized, particularly in Western countries. Many non-European/North American countries lack data regarding the prevalence and impacts of sleep problems [4]. Although there is growing evidence on the public health importance of sleep disorders in developing countries, studies on this issue are scarce. Available data suggest that sleep problems are highly prevalent, sleep awareness is limited, and sleep disorders represent an insufficiently recognized public health issue in those countries [5,6]. Likewise, sleep medicine development in many former Soviet Union countries remains at low levels, the importance of sleep for health and quality of life is often neglected and the majority of individuals with sleep disorders are not diagnosed and treated [4].

Quality of life is a multidimensional concept encompassing the physical, psychological, and social domains [7], all of which may be influenced by sleep. A variety of sleep disorders can aggravate medical problems and compound impairments in health-related quality of life (HRQoL) domains [8]. Furthermore, sleep disturbances may worsen quality of life independently of their impact on physical and mental well-being [3]. While several types of sleep disorders have been reported to influence HRQoL [9,10,11,12], research on this issue in the general population remains limited. Moreover, the association between sleep disorders and HRQoL is less clear in the developing world, where socioeconomic and political changes and accelerating trends towards urbanization may be associated with an increased risk of poor health, social instability, and sleep problems [4,13]. Indeed, to our knowledge, up to now, there is no study focused specifically on the assessment of the association between sleep and HRQoL in individuals from developing countries—populations with different sociocultural and environmental background compared to Western societies; little has been known about the prevalence of sleep disorders in the developing world. Therefore, the Georgia SOMNUS Study was conducted in Georgia, one of the former Soviet Union Country and an underevaluated culture with regard to sleep care, as the case with many countries from the developing world [4]. Without any published evidence on the prevalence of sleep disorders in the country, the overall aim of the Georgia SOMNUS Study was to provide subjective data on sleep–wake behavior and sleep quality, on the prevalence of sleep disorders and associated factors, to focus on the relationship between sleep and HRQoL, and to examine results in light of the data from Western societies. Previous publication from the Georgia SOMNUS study reported data on sleep–wake pattern and multiple dimensions of sleep quality [4], while the present study aimed to explore the prevalence and severity of various sleep disorders and their associations with HRQoL in an urban Georgian population after adjusting for sociodemographic and health variables.

## 2. Methods

A complete description of the study and its methods has been published elsewhere [4] and is briefly summarized here. The cross-sectional Georgia SOMNUS Study was carried out in April–May 2013 and included 395 unpaid volunteers aged between 20 and 60 years. The sample consisted of residents of Tbilisi (the capital) and Kutaisi (the second-largest city in Georgia). Interviews were conducted to collect sociodemographic information such as age, sex, marital status (married/cohabiting or single/divorced/widowed), educational level (high school, college, or university), current employment status (employed/unemployed), and self-rated economic status based on household income (rated from 1 (very bad) to 5 (very good)). In addition, the subjects reported their height and weight to calculate body mass index (BMI). As part of this study, several health- and sleep-related questionnaires were administered (described in detail below). Subjects unable to complete the interview and fill out the questionnaires due to medical reasons (e.g., cognitive impairment) were excluded from the study. Informed consent was obtained from all participants. The study was conducted in accordance with the Declaration of Helsinki, and the protocol was approved by the Ethics Committee of Ilia State University, Tbilisi, Georgia.

## 3. Measures

The Pittsburgh Sleep Quality Index (PSQI) is one of the most widely used questionnaires to assess sleep quality [14]. The PSQI examines sleep over the past month and consists of 19 self-rated items grouped into the 7 component scores: subjective sleep quality index, sleep latency, duration, efficiency, sleep disturbances, use of sleeping medication, and daytime dysfunction. Each component is scored on a four-point scale (0–3). The sum of the seven components results in a Global PSQI score ranging from 0 (high sleep quality) to 21 (low sleep quality). A global PSQI score > 5 is considered indicative of poor sleep quality. In this study, the Cronbach’s α, a measure of internal consistency, of the PSQI seven components score was 0.77.

The Beck Depression Inventory-Short Form (BDI–SF) is a 13-item subset of the original instrument used to assess depressive symptoms experienced over the past week based on a four-point scale [15]. Higher scores suggest more severe depressive symptomatology. The BDI has been used in previous studies of the Georgian population [16,17].

The Insomnia Severity Index (ISI) is a seven-item questionnaire that has been widely used as a subjective index of sleep difficulties [18]. Items are evaluated on a five-point scale (0 = not at all, 4 = extremely) to yield a total score ranging from 0 to 28. Scores are classified into four severity categories: absence of insomnia (0–7), subthreshold (8–14), moderate (15–21), and severe (22–28) insomnia. The Georgian version of the questionnaire has been found to have good test–retest reliability [16]. In this study, the Cronbach’s α of the ISI-7 components score was 0.89.

The Epworth Sleepiness Scale (ESS) is an eight-item questionnaire designed to determine the likelihood of dozing in eight different situations commonly encountered in daily life [19]. Each item is rated from 0 (no chance of dozing) to 3 (high chance of dozing). Thus, the total score of the ESS ranges from 0 to 24. Higher scores indicate higher levels of excessive daytime sleepiness (EDS). A global score > 10 is used to identify individuals with EDS. The Cronbach’s α was 0.76.

The STOP-Bang questionnaire [20] is an 8-item screening tool developed to identify subjects with a probability of obstructive sleep apnea (OSA). The total score ranges from 0 to 8. A score ≥ 3 is widely used for OSA screening. More recently, the following STOP-Bang scores have been proposed: a score of 0–2 indicates a low risk of OSA; 3–4 indicates an intermediate risk; and a score of 5–8 indicates a high risk of OSA [21].

HRQoL was assessed using the short-form SF-12 Health Survey, version 2 [22]. The SF-12 is a short-form of the SF-36, the most widely used health survey, and this version has been found to avoid any substantial loss of information relative to the use of the SF-36 [23]. The SF-12 consists of physical and mental component summary (PCS and MCS) measures and eight health domain scores (physical functioning, physical role, bodily pain, general health, vitality, social functioning, emotional role, and mental health). Higher scores on these subscales suggest better quality of life. The SF quality of life survey has been widely used in Georgian population [24,25]. In this study, the Cronbach’s α of the SF-12 eight components score was 0.85. Unadjusted SF-12 domain scores were used for analysis.

Although neither the SF-36 nor the SF-12 includes items specific to sleep [3,26], both instruments have been extensively used to assess the association between HRQoL and sleep disorders.

### 3.1. Sleep Status Groups Classification

The participants were classified according to their sleep problems. For overall sleep quality we separated the sample into ‘good sleepers’ and ‘poor sleepers’ (PSQI cut-off score > 5).

For the daytime sleepiness, we identified groups of subjects with and without EDS based on a cut-off ESS score > 10.

Insomnia was categorized as follows. First, the subjects were separated into those with any degree of insomnia (subthreshold, moderate or severe; ISI score 8–28) and those without insomnia (ISI score 0–7). Second, ISI scores were used to separate the subjects into four insomnia severity groups (no insomnia, subthreshold, moderate, and severe insomnia).

Similarly, the risk of OSA was categorized as follows. First, subjects with and without apnea risk were separated using a STOP-Bang cut-off score of ≥3; Second, we examined a higher threshold of apnea risk using a cut-off score ≥ 4. Third, the subjects were categorized into the following three groups: low risk (score 0–2), intermediate risk (score 3–4), and high risk of OSA (score 5–8).

### 3.2. Statistical Analysis

Sociodemographic, sleep, and health variables are described using counts and percentages for categorical variables and means and standard deviations for continuous variables. Differences in means between the sleep status groups were assessed using *t*-test for normally distributed variables or the Mann–Whitney U test for non-normally distributed variables. The normality of the variables were tested by the Shapiro–Wilk test. Differences in the means between the sleep status groups were assessed using *t*-test or the Mann–Whitney U test, as appropriate. For non-parametric tests, medians and interquartile ranges are reported. Differences in SF-12 scores across insomnia severity (no insomnia, subthreshold, moderate, and severe insomnia) and apnea-severity groups (low, intermediate, and high risk of OSA) were assessed using analysis of variance (ANOVA). The relationship between the severity of sleep problems (insomnia, apnea risk) and HRQoL was analyzed using Spearman correlation coefficients.

To estimate the clinical meaningfulness of differences in SF-12 summary scores between subjects with and without sleep disorders, comparisons were presented using effect size and 95% confidence interval statistics [27]. Building on similar studies for the interpretation of results, we present the raw differences between group means (absolute effect size) and 95% confidence intervals [28]. Given that US-derived summary scores with a mean of 50 and standard deviation of 10, have been suggested for cross-cultural comparisons of results [23,29], an effect size of 2 points with a 95% confidence interval that does not include 0 would be considered as a small, yet clinically meaningful effect size [28].

In addition, to determine the strength of the association between sleep quality and HRQoL, hierarchical multiple linear regression analyses were performed with age, sex, marital status, BMI, education, employment status, economic status, and BDI scores in Step I and the components of Step I plus global PSQI score in Step II. Analyses were performed individually for each of the SF-12 components and summary scores, which were examined as outcome variables, thus yielding 10 sessions of hierarchical regression. Similarly, separate hierarchical regression analyses were performed using the SF-12 PCS and MCS scores as outcome variables and the ESS, ISI, or STOP-Bang scores as predictor variables, which were added individually one by one, in Step II. Thus, yielding six sessions of hierarchical regression—three for PCS and three for MCS. All categorical variables were dummy coded, and all regression models were checked for multicollinearity with a variance inflation factor (VIF). The VIF in our models were below 2 for all variables. In addition, a normal predicted probability (P-P) plots and the scatterplots of the residuals and predicted values were examined to check for a normal distribution and homoscedasticity. All models met the associated assumptions. Regression coefficients with 95% confidence intervals and both R^2^ and the adjusted R^2^ values are reported. A two-sided significance level was set at α = 0.05. All analyses were performed using IBM SPSS Statistics 21.0 (IBM Corp; Armonk, NY, USA, 2012).

## 4. Results

### 4.1. Characteristics of Study Participants

Table 1 presents the demographic and health characteristics of the studied population. As reported in a previous publication from the Georgia SOMNUS Study [4], the mean age of the participants was 37.7 years (SD = 10.8). Most of the subjects were female (67.6% vs. 32.4%). The majority of subjects had a university degree (80.8%), were married or cohabiting (68.4%) and were employed (68.9%). The economic status of the participants varied widely from very bad (3.3%) to very good (9.1%), with the majority of participants demonstrating an average status (45.6%).

Forty-three percent of the studied population reported poor sleep quality (PSQI > 5). Daytime sleepiness was observed in 17% of the subjects. A risk of OSA (assessed by STOP-Bang ≥ 3) was identified in 34.2% of the population. In the analysis of risk severity, 27.4% of the participants presented an intermediate risk of sleep apnea, and 6.8% of the participants had a high risk. A strong positive correlation was identified between the ESS and STOP-Bang scores (r = 0.6, *p* < 0.001).

The percentage of subjects with EDS (17%) was lower than the proportion of subjects at risk of sleep apnea when a cut-off value ≥ 3 was applied (34.2%). Nevertheless, when the distribution of ESS scores across STOP-Bang scores was assessed, the mean ESS score among subjects with three positive answers on the STOP-Bang was 8.2 (normal range of ESS), and those subjects constituted 16.7% of the studied population. The mean ESS score among subjects with four positive answers on the STOP-Bang was 10.1 (range indicating EDS), and those subjects constituted 10.6% of the studied population.

Overall, 32.9, 9.4, and 1.8% of the population reported subthreshold insomnia, moderate insomnia, and severe insomnia, respectively.

### 4.2. Health-Related Quality of Life

To determine whether quality of life in several health domains differed in association with sleep problems, SF-12 measures, BDI–SF scores, and BMI values were compared between groups defined by sleep quality (good vs. poor), daytime sleepiness status, OSA risk and insomnia status (all with vs. without; Table 2). The SF-12 component scores were significantly lower among those with poor sleep quality than those with good sleep quality. A significant difference was identified for BDI–SF score but not for BMI value. Similarly, subjects with and without daytime sleepiness differed significantly on all SF-12 measures as well as by BDI–SF score and BMI value. Similar findings were found for insomnia, although no significant between-group difference was identified for BMI. The pattern of differences was the same for PCS and MCS scores (Table 3). The reduction was statistically significant in all but one case (MCS among participants with OSA risk). When sleep apnea was defined using a cut-off score of ≥3, the groups did not differ on the following three SF-12 subscales: vitality, social functioning, and MCS. The use of a higher threshold of apnea risk (≥4) revealed significant differences in all SF-12 component and summary scores (Table 3).

The effect sizes for differences in mean scores of PCS and MCS scores between sleep status groups demonstrated that there was a small but clinically meaningful reduction in physical and mental quality of life associated with having a poor sleep quality, EDS, OSA risk or insomnia, except for MCS score in subjects with and without OSA risk (Table 3).

**Figure 1:**
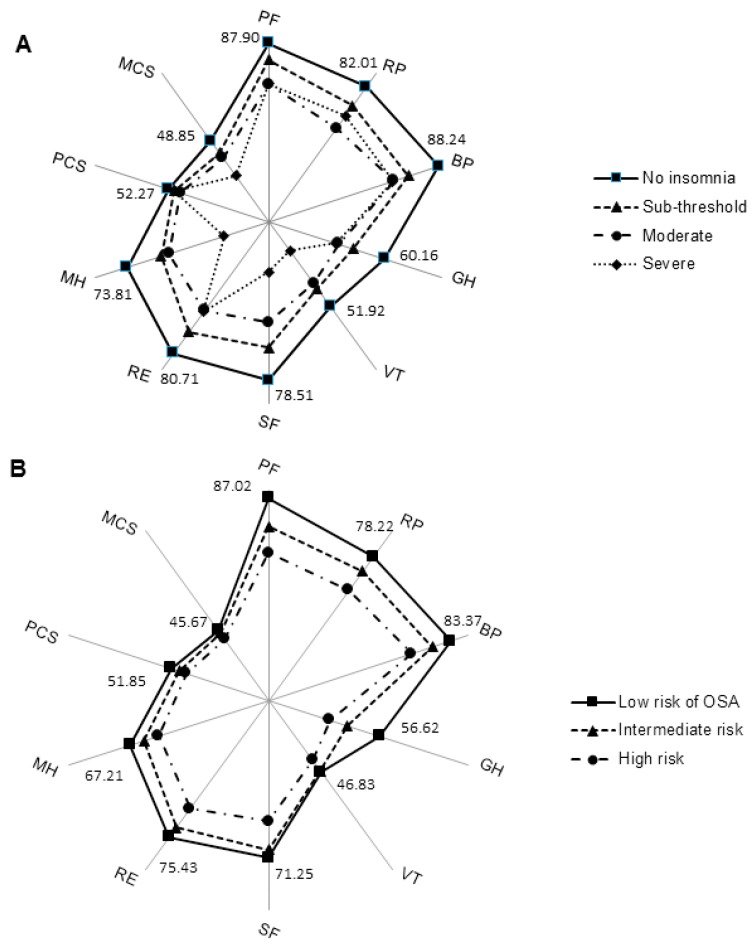
The mean values of the different SF-12 subscales across insomnia-severity groups (**A**) and apnea-severity groups (**B**). PF—physical functioning; RP—physical role; BP—bodily pain; GH—general health; VT—vitality; SF—social functioning; RE—emotional role; MH—mental health; PCS—physical component summary; MCS—mental component summary; OSA—obstructive sleep apnea.

We also evaluated whether insomnia or apnea severity were correlated with HRQoL components. A negative correlation was identified between insomnia severity and HRQoL, and while insomnia severity was significantly correlated with all SF-12 scales and summary scores, the highest correlation was observed for MCS score (r_s_ = −0.43, *p* < 0.001). The correlation between apnea risk severity and HRQoL was significant for all scales except vitality and social functioning. OSA severity was most highly correlated with PCS (r_s_ = −0.27, *p* < 0.001). Indeed, as shown on Figure 1A, the mean values of the SF-12 subscales showed incrementally lower values in most SF-12 dimensions across insomnia severity groups. The most noticeable reduction were identified in the vitality, social functioning, mental health, and mental component summary scores. Similarly, the increased risk of OSA was associated with poorer HRQoL on most domains of the SF-12 (Figure 1B). Although the scores were lower, no statistically significant between group differences in vitality (*p* = 0.57), social functioning (*p* = 0.08), or MCS (*p* = 0.16) scores were observed for OSA severity.

### 4.3. Association of Sleep Disorders with Health-Related Quality of Life

To analyze the impact of sleep problems on HRQoL, we performed separate hierarchical regression analyses for the associations between sleep quality, which addresses multiple dimensions of sleep, and each of the SF-12 component and summary scores individually as the dependent variables. Hierarchical regression analysis showed that poor sleep quality remained a significant predictor of each of the SF-12 scales, even after adjustments for demographic variables, BMI, and BDI. Specifically, the addition of the PSQI global score during the second step of analysis significantly increased the model’s predictive ability, with an R^2^ change (ΔR^2^) of 3.5% identified for PCS and 2.9% identified for MCS; for the other SF-12 component scores, increases in predictive power ranged between 1.4% and 4.6% (Table 4). In addition, the only variable that remained significantly associated with all SF-12 subscales and PCS and MCS scores in final models was the depression score.

We also examined the association of the ESS, STOP-Bang, and ISI scores with the SF-12 PCS and MCS domains using separate hierarchical regression models. Six models were developed wherein PCS or MCS was the dependent variable, demographic variables, BMI, and BDI scores were entered in Step I, while ESS, STOP-Bang, or ISI scores were added individually in Step II (see Supplementary Table S1). In all analyses, we found small but statistically significant changes in the model’s predictive power. The greatest effect was observed for the association between ISI and MCS (ΔR^2^ = 0.039, *p* < 0.001, β = −0.24). For all sleep variables examined, depression was significantly associated with both the MCS and PCS scores, while economic status was significantly associated only with the MCS. As regards BMI, it remained a significant predictor in the association between PCS and all sleep variables, and between MCS and STOP-Bang scores.

## 5. Discussion

Available data suggest that sleep disorders are associated with impairment of HRQoL. However, to the best of our knowledge, this is the first study to assess the association of different sleep disorders with HRQoL in a developing country. We found that the prevalence of sleep disorders was high in an urban Georgian population sample. Some degree of insomnia was identified in 44.1% of the sample. However, only 11.2% scored in the moderate-to-severe insomnia range, while 32.9% of the studied population scored in the subthreshold range for insomnia. It has become widely accepted that the prevalence of insomnia varies depending on its definition, the methodology used and the population studied. In general, insomnia is present as a symptom in approximately 30–50% of the adult population, and is present as a clinical syndrome in 5–10% [30]. Although a clinical assessment of insomnia was not performed in the current study, we evaluated insomnia based on the ISI score, which has been deemed a reliable instrument for insomnia detection. ISI scores corresponding to moderate-to-severe insomnia may be interpreted as the presence of clinically significant insomnia, and the prevalence of such scores in our sample (11.2%) is well aligned with the results of previous epidemiological studies [30]. Nevertheless, given that a cut-off score of 10 from subthreshold range has been suggested to be preferable for identifying insomnia cases in the general population [31], the prevalence of insomnia cases in our sample (27.8% with a cut-off score of 10) is higher than that reported in the general population from Western countries [32,33].

EDS is a distinctive symptom of several sleep disorders. The prevalence of EDS in the current study was 17%, which is slightly higher than that reported in the literature [34,35]. EDS is considered an important complaint among those with sleep-disordered breathing. However, the mechanism underlying EDS is multifactorial, and in addition to sleep problems, a variety of other factors have been reported to be associated with EDS [34,36]. In the present study, we did not identify the determinants of EDS. However, analyzing the distribution of ESS scores across STOP-Bang scores showed that subjects at sleep apnea risk with three positive answers on the STOP-Bang may have normal range of ESS, not indicative of EDS. Thus, our data support the hypothesis that the prevalence estimates for no, mild, or moderate sleep apnea among those with STOP-Bang scores of 3 and 4 may be evenly balanced [37] and that STOP-Bang threshold at 4 may be more useful in the general population [38]. This may partially explain a somewhat higher rate of sleep apnea with a cut-off value at ≥3 (34.2%) in our study population compared to other studies [39].

One of the major objectives of this study was to address the association between different sleep disorders and HRQoL. The current study corroborates and builds upon evidence suggesting the presence of a link between sleep and HRQoL. We observed that there were a clinically meaningful reduction in all SF-12 components and summary scores among subjects with sleep disorders, and similar results were identified for poor sleep quality, EDS, insomnia, and apnea risk (cut-off ≥ 4). A clear definition of a clinically meaningful change in HRQoL research has not yet been established. A1-point lower score on SF-36 selected scales was found to be associated with an excess risk of up to 9% for mortality and 12% for work inability in patients with diabetes [40]. HRQoL research has often focused on the concept of a minimally important difference (MID) to provide benchmarks for the interpretation of changes. General recommendations for MIDs range from 2 to 4 points to be considered clinically important [41]. A difference of five points in SF-12 summary scores, a common MID threshold, has also been suggested as a meaningful [26,42]. Therefore, interpretations are difficult when an observed difference is smaller but statistically significant [43]. Effect size estimates provided for use in HRQoL racial/ethnic sleep studies for physical health were 1.5 and 2.9 points in participants with and without EDS and insomnia, and for mental health −2 and 2.7 points, respectively [28]. Thus, our findings suggest that the differences in HRQoL between subjects with and without poor sleep quality, EDS, apnea risk, or insomnia, are clinically meaningful.

Furthermore, we found that the majority of the SF-12 scales were sensitive to sleep-disorder severity (insomnia severity, apnea risk severity). More severe sleep problems appeared to be associated with poorer HRQoL. This finding is in accordance with those of other studies showing that insomnia severity or the severity of sleep-disordered breathing is associated with worsened quality of life [9,44,45]. In addition, the impact of insomnia severity on the SF-12 scales was more pronounced for the mental component variables. Other studies have also reported that insomniacs scored lower or the same as non-insomniacs in the physical components of HRQoL [31,46].

In the present study, we also observed that controlling for depression, sociodemographic factors, and BMI still resulted in the identification of a statistically significant association between sleep quality and the HRQoL dimensions, and between the other evaluated types of sleep disorders (daytime sleepiness, insomnia, and sleep apnea risk) and MCS and PCS scores. The association of sleep disorders with HRQoL outcomes underscores the need to examine socioeconomic status, cultural factors, health covariates, and other factors known to influence HRQoL [28,34]. Since some potential confounders have been tested in our regression analysis, small effect sizes found in the current study are likely to have clinical importance.

Our findings are somewhat different from published results. Although other studies have reported the presence of significant associations between HRQoL and OSA, insomnia, or daytime sleepiness, associations were present mainly for severe sleep problems and not for all SF-12 scales [44,45,47]. We can hypothesize that the association between poor sleep and all aspects of HRQoL observed herein may be somehow influenced by some general sociological factors (e.g., changes in the country’s political situation in the last decades, ethnic conflicts, accelerating trends toward urbanization) resulting in an increased risk of psychosocial instability. This hypothesis is in line with the data reporting the negative impact of low socioeconomic status on sleep and health [48,49]. The results of some of these studies suggest that sleep may serve as a mediating factor in the association between socioeconomic status and poor health [48]. In the previous study of this sample, we showed that economic status was the most significant predictor of poor sleep [4]. In this study, we found that economic status was significantly associated with the mental component summary scores for all sleep variables. A negative association between socioeconomic status and HRQoL have also been shown in a number of studies [50,51]. While economic status and depression may contribute to poor sleep, sleep problems appeared to be independent predictors of poorer quality of life in the current analyses. We cannot assess how much the economic patterning of sleep explains the gradient in the impact of sleep on HRQoL. However, this finding, along with the fact of economic status being statistically the most significant predictor of poor sleep, emphasizes the importance of carefully investigating sleep problems and their impact on HRQoL in individuals of low socioeconomic status.

With respect to BMI and depression, our findings correspond to the previous literature, which described the strong associations between depression and SF-12 MCS scale, and BMI and SF-12 PCS scale [52,53,54]. Also similar to our results, the association of BMI and some of mental component dimensions of HRQoL were found in OSA patients [55]. Evidence indicate that BMI and depression may contribute to sleep difficulties [34,39,56]. Our data also showed that depression symptoms were significantly higher in subjects with poor sleep quality, daytime sleepiness, OSA risk, and insomnia, which was to be expected. The significant difference for BMI was found between groups defined by daytime sleepiness and OSA risk (with vs. without). However, after further consideration of these variables using hierarchical regression models, we found that none of the variables (demographics, BMI, depression) had a variance inflation factor greater than 2, and the addition of the sleep variables, one by one, significantly improved prediction in all models. Therefore, our data provide evidence that subjective sleep symptoms are comprehensively associated with impairment in HRQoL. Sleep disorders are a major problem worldwide, but their prevalence and the extent to which HRQoL is influenced by sleep is poorly documented in the developing world, which is an apparent need of greater attention to sleep care.

Some limitations of this study should be acknowledged. First, the cross-sectional nature of the study (data collected on the study population at a single time point) made it impossible to establish a causal relationship between sleep disturbances and HRQoL. Second, data were based on self-reported questionnaires and objective sleep measurements were not performed. Furthermore, PSQI and SF-12 contain a few seemingly similar questions that may influence study results. However, we analyzed PSQI global score, a global measure of sleep quality based on seven components scores, which minimizes such influence on study findings. The study is also limited by the unequal sex distribution that could potentially impact the prevalence rate of sleep disorders. Future examination of these data with a stratified analysis will be addressed in a separate paper. A further limitation is that some lifestyle factors, medical issues, and psychiatric disorders (other than depression) that may affect HRQoL were not directly assessed in the present study. It is also important to consider that physical and mental health problems, often associated with sleep disorders, may have been confounding factors in the observed associations. However, exclusion of subjects with severe health conditions limits the influence of this factor on study results. Furthermore, it is also possible that health problems worsen sleep disorders and thereby trigger worse HRQoL. In this study we did not investigate the causes of sleep disorders. However, given that after controlling for potential confounders (demographic variables, BMI, and depression), all sleep disorders showed clinically meaningful associations with HRQoL, we may say that a clear relationship exists between sleep and HRQoL.

The distinctive strengths of this study should also be mentioned. Data were collected using standardized, widely used measures. In addition, the majority of subjects had not undergone treatment for their sleep disorders [4]. Moreover, the relationship between sleep and HRQoL was assessed across different sleep disorder groups (insomnia, daytime sleepiness, sleep apnea risk, sleep quality) in the same population sample that included both good and poor sleepers, subjects with and without sleep disorders, which strengthens the observed associations.

## 6. Conclusions

The high prevalence of sleep disorders observed in the present study calls for more comprehensive assessments of sleep disorders in Georgia and more globally, in countries where the clinical significance of sleep disorders and their burden is not well recognized. Furthermore, these data confirm and extend observations from Western societies to the developing world and strongly support the importance of sleep for health and well-being. This link is especially important in low-income settings, where the impact of economic status on the burden of sleep problems and thereby on HRQoL is likely to be considerable. From a public health perspective, intervention programs designed to strengthen sleep-related health care in developing countries where people do not often report their sleep complaints to medical practitioners and may not even be aware that sleep disorders are significant health concerns with clear impact on HRQoL is a global healthcare challenge that must be addressed.

## Figures and Tables

**Table 1:** Counts and percentages (**A**), and means and standard deviations (**B**) for demographic and health characteristics of the study population.

(**A**)		
	***N***	**%**
**Sex**		
Female	267	67.6
Male	128	32.4
**Marital status**		
Married/cohabiting	270	68.4
single/divorced/widowed	125	31.6
**Education**		
High school	32	8.1
College	44	11.1
University	319	80.8
**Employment status**		
Employed	272	68.9
Unemployed	123	31.1
**Economic status**		
Very bad	13	3.3
Bad	60	15.2
Average	180	45.6
Good	106	26.8
Very good	36	9.1
**Daytime sleepiness**		
No sleepiness (ESS score ≤ 10)	328	83
Sleepiness (ESS score > 10)	67	17
**OSA risk**		
Low risk (STOP-Bang score 0–2)	260	65.8
Intermediate (STOP-Bang score 3–4)	108	27.4
High risk (STOP-Bang score 5–8)	27	6.8
**Insomnia severity**		
No insomnia (ISI score 0–7)	221	55.9
Subthreshold (ISI score 8–14)	130	32.9
Moderate (ISI score 15–21)	37	9.4
Severe (ISI score 22–28)	7	1.8
**Sleep quality**		
Good (PSQI score ≤ 5)	225	57
Poor (PSQI score > 5)	170	43
(**B**)		
	**Mean**	**SD**
**Age (years)**	37.7	10.8
**BMI**	25.3	5.2
**BDI–SF**	4.2	4.3
**HRQoL**		
Physical functioning	83.0	24.1
Physical role	75.5	22.4
Bodily pain	80.4	23.2
General health	52.1	25.7
Vitality	46.3	23.9
Social functioning	69.6	29.0
Emotional role	73.5	23.4
Mental health	65.0	23.1
	**Mean (Range)**	**SD**
**PCS**—Physical component summary	50.6 (22.2–70.1)	6.9
**MCS**—Mental component summary	45.2 (11.6–67.6)	9.9

OSA—obstructive sleep apnea; BMI—body mass index; BDI–SF—Beck depression inventory–short form; HRQoL—health-related quality of life.

**Table 2:** Differences in the SF-12 variables according to sleep status groups.

SF-12	Sleep Quality—PSQI (Mean, SD)		Daytime Sleepiness—ESS (Mean, SD)		OSA Risk—STOP-Bang (Mean, SD)		Insomnia—ISI (Mean, SD)	
	Score ≤ 5	Score > 5	Score ≤ 10	Score > 10	Score < 3	Score ≥ 3	Score ≤ 7	Score > 7
PF	88.0 (21.5)	76.3 (25.7) ***	84.9 (22.7)	73.1 (27.9) **	87.0 (20.9)	75.2 (27.7) ***	87.9 (22.2)	76.7 (25.0) ***
RP	81.9 (19.9)	66.9 (22.8) ***	77.2 (21.3)	66.9 (25.5) **	78.2 (21.5)	70.3 (23.3) **	82.0 (20.1)	67.2 (22.5) ***
BP	88.0 (17.5)	70.4 (25.9) ***	82.0 (21.9)	72.8 (27.4) **	83.4 (21.6)	74.8 (25.1) ***	88.2 (17.8)	70.5 (25.5) ***
GH	59.9 (23.3)	41.6 (25.0) ***	53.7 (25.2)	44.0 (26.8) **	56.6 (24.4)	43.3 (26.0) ***	60.2 (23.2)	41.8 (25.1) ***
VT	52.0 (22.9)	38.8 (23.1) ***	47.4 (23.9)	41.0 (22.9) *	46.8 (24.7)	45.4 (22.3)	51.9 (23.2)	39.2 (22.9) ***
SF	78.3 (25.0)	58.1 (29.9) ***	71.2 (28.4)	61.9 (30.6) *	71.3 (28.4)	66.5 (29.9)	78.5 (25.6)	58.3 (29.1) ***
RE	80.8 (19.7)	63.8 (24.5) ***	74.8 (23.3)	66.8 (22.9) *	75.4 (22.9)	69.7 (24.1) *	80.7 (19.3)	64.3 (24.9) ***
MH	73.5 (17.9)	53.8 (24.3) ***	66.3 (23.2)	58.8 (21.4) *	67.2 (23.2)	60.8 (22.2) **	73.8 (18.3)	53.9 (23.7) ***
BDI–SF ^	2.0 (4.0)	5.0 (8.0) ***	3.0 (4.0)	4.0 (6.0) **	3.0 (5.0)	4.0 (5.0) **	2.0 (4.0)	5.0 (8.0) ***
BMI ^	24.2 (7.0)	24.9 (6.6)	24.2 (6.5)	27.4 (8.6) ***	23.1 (5.8)	27.9 (7.8) ***	24.7 (7.0)	24.5 (6.5)

*—*p* < 0.05; **—*p* < 0.01; ***—*p* < 0.001; PF—physical functioning; RP—physical role; BP—bodily pain; GH—general health; VT—vitality; SF—social functioning; RE—emotional role; MH—mental health; BDI–SF—Beck depression inventory–short form; BMI—body mass index. ^—Data are presented as the median and interquartile range. Statistics are based on the Mann–Whitney test for BDI–SF and BMI, and on *t*-test for SF-12 variables.

**Table 3:** Physical and mental component summary scores and effect sizes according to sleep status groups.

SF-12	Physical Component Summary—PCS		Mental Component Summary—MCS	
	(Mean, SD)	Effect Size (95% CI)	(Mean, SD)	Effect Size (95% CI)
**Sleep quality—PSQI**				
Good (score ≤ 5)	52.3 (5.8)	3.9 (2.5–5.3) ***	48.8 (8.2)	8.4 (6.5–10.3) ***
Poor (score > 5)	48.3 (7.6)		40.4 (10.1)	
**Excessive daytime sleepiness—EDS**				
No (score ≤ 10)	51.2 (6.6)	3.5 (1.7–5.3) ***	45.7 (10.0)	2.9 (0.3–5.5) *
Yes (score > 10)	47.7 (7.6)		42.7 (9.1)	
**OSA risk—STOP-Bang**				
No (score < 3)	51.8 (6.4)	3.7 (2.3–5.1) ***	45.7 (10.0)	1.5 (−0.6–3.5)
Yes (score ≥ 3)	48.1 (7.2)		44.2 (9.7)	
No (score < 4)	51.3 (6.7)	4.1 (2.4–5.9) ***	46 (9.8)	4.7 (2.2–7.3) ***
Yes (score ≥ 4)	47.2 (6.9)		41.3 (9.6)	
**Insomnia—ISI**				
No (score ≤ 7)	52.3 (5.8)	3.9 (2.5–5.2) ***	48.8 (8.1)	8.4 (6.5–10.2) ***
Yes (score > 7)	48.4 (7.6)		40.5 (10.1)	

*—*p* < 0.05; ***—*p* < 0.001; CI—confidence interval.

**Table 4:** Hierarchical multiple regression analyses of the associations between sleep quality and HRQoL scales.

Outcome Variables	Step I			Step II (Variables in Step I plus PSQI Global Score)					
	F(_12,382_)	R^2^	Adjusted R^2^	ΔF(_1381_)	Adjusted R^2^	ΔR^2^	B for PSQI	β	95% CI
PF	7.69 ***	0.19	0.17	11.71 **	0.19	0.024	−1.43	−0.19	−2.25/−0.61 **
RP	12.45 ***	0.28	0.26	10.52 **	0.28	0.019	−1.19	−0.17	−1.9/−0.47 **
BP	12.15 ***	0.28	0.25	25.69 ***	0.30	0.046	−1.90	−0.26	−2.6/−1.16 ***
GH	16.52 ***	0.34	0.32	28.77 ***	0.37	0.046	−2.11	−0.26	−2.89/−1.34 ***
VT	7.5 ***	0.19	0.17	12.04 **	0.19	0.025	−1.44	−0.19	−2.25/−0.62 **
SF	16.57 ***	0.34	0.32	8.25 **	0.33	0.014	−1.31	−0.14	−2.20/−0.41 **
RE	12.14 **	0.28	0.25	16.64 ***	0.28	0.03	−1.56	−0.21	−2.31/−0.81 ***
MH	29.74 ***	0.48	0.47	23.06 ***	0.50	0.03	−1.51	−0.21	−2.13/−0.89 ***
PCS	11.05 **	0.26	0.23	18.75 ***	0.27	0.035	−0.49	−0.23	−0.72/−0.27 ***
MCS	28.21 **	0.47	0.45	21.88 ***	0.48	0.029	−0.64	−0.21	−0.91/−0.37 ***

PF—physical functioning; RP—physical role; BP—bodily pain; GH—general health; VT—vitality; SF—social functioning; RE—emotional role; MH—mental health; PCS—physical component summary; MCS—mental component summary. Variables in Step I: age, sex, marital status, body mass index, employment status, economic status, education, and depression score. F—test of overall model significance; R^2^—coefficient of determination; B—regression coefficient; β—standardized regression coefficient; ΔF—F change; ΔR^2^—change in R^2^ value; CI—confidence interval; **—*p* < 0.01; ***—*p* < 0.001

## Supplementary Materials

The following are available online at http://www.mdpi.com/1660-4601/15/8/1588/s1. Table S1: Hierarchical multiple regression analyses of the associations between daytime sleepiness (A), OSA risk (B) and insomnia (C) and HRQoL summary scores.
