# Supplementary material for: Relationship between Sleep Disorders and Health Related Quality of Life—Results from the Georgia SOMNUS Study
Source: Int J Environ Res Public Health. 2018 Jul 26;15(8):1588. doi: 10.3390/ijerph15081588 (PMC6121956; doi:10.3390/ijerph15081588)
Supplement: Supplementary file 1 [file ijerph-15-01588-s001.pdf]

Supplementary Table 1. Hierarchical multiple regression analyses of the associations between daytime sleepiness (A), OSA risk (B) and insomnia (C) and HRQoL summary scores

A

Outcome Variables	Step I		Step II (Variables in Step I plus ESS)				
	$F_{(12,382)}$	R^2	$\Delta F_{(1,381)}$	ΔR^2	B for ESS	95% CI	β
PCS - Physical component summary	11.05***	0.26	9.76**	0.019	-0.28	-0.45 / -0.10**	-0.15
MCS - Mental component summary	28.21***	0.47	7.2**	0.01	-0.29	-0.50 / -0.08**	-0.11

B

Outcome Variables	Step I		Step II (Variables in Step I plus STOP-Bang Score)				
	$F_{(12,382)}$	R^2	$\Delta F_{(1,381)}$	ΔR^2	B for STOP-Bang	95% CI	β
PCS - Physical component summary	11.05***	0.26	4.9*	0.009	-0.62	-1.17 / -0.07*	-0.14
MCS - Mental component summary	28.21***	0.47	6.59*	0.009	-0.87	-1.54 / -0.20*	-0.14

C

Outcome Variables	Step I		Step II (Variables in Step I plus ISI Score)				
	$F_{(12,382)}$	R^2	$\Delta F_{(1,381)}$	ΔR^2	B for ISI	95% CI	β
PCS - Physical component summary	11.05***	0.26	11.97**	0.023	-0.23	-0.36 / -0.10**	-0.23
MCS - Mental component summary	28.21***	0.47	29.88***	0.039	-0.43	-0.58 / -0.27***	-0.24

ESS - Epworth sleepiness scale, ISI - insomnia severity Index. Variables in Step I: age, sex, marital status, body mass index, employment status, economic status, education, and depression score. F - test of overall model significance; R^2 - coefficient of determination; ΔF - F change; ΔR^2 – change in R^2 value; B - unstandardized regression coefficient; CI - confidence interval; β - standardized regression coefficient; * - $p<0.05$; ** - $p<0.01$, *** - $p<0.001$
